# Use of Smartphone Apps, Social Media, and Web-Based Resources to Support Mental Health and Well-Being: Online Survey

**DOI:** 10.2196/12546

**Published:** 2019-07-12

**Authors:** Katarzyna Stawarz, Chris Preist, David Coyle

**Affiliations:** 1 Bristol Interaction Group Faculty of Engineering University of Bristol Bristol United Kingdom; 2 School of Computer Science University College Dublin Dublin Ireland

**Keywords:** mental health, mobile apps, mHealth, social media, self-instruction programs, computerized

## Abstract

**Background:**

Technology can play an important role in supporting mental health. Many studies have explored the effectiveness, acceptability, or context of use of different types of mental health technologies. However, existing research has tended to investigate single types of technology at a time rather than exploring a wider ecosystem that people may use. This narrow focus can limit our understanding of how we could best design mental health technologies.

**Objective:**

The aim of this study was to investigate which technologies (smartphone apps, discussion forums and social media, and websites and Web-based programs) people use to support their mental health and why, whether they combine and use more than one technology, what purpose each technology serves, and which features people find the most valuable.

**Methods:**

We conducted an online survey to gather responses from members of the public who use technology to support their mental health and well-being. The survey was advertised on social media and via posters at a university. It explored usage patterns, frequently used features, and engagement with technology. To gain deeper insights into users’ preferences, we also thematically analyzed open-ended comments about each technology type and suggestions for improvements provided by the respondents.

**Results:**

In total, 81 eligible participants completed the survey. Smartphone apps were the most commonly used technology, with 78% of the participants (63/81) using them, either alone (40%) or in combination with other technologies (38%). Each type of technology was used for specific purposes: apps provided guided activities, relaxation, and enabled tracking; social media and discussion forums allowed participants to learn from the experiences of others and use that knowledge to understand their own situation; and Web-based programs and websites helped to find out how to deal on a day-to-day basis with stress and anxiety. The analysis of open-ended responses showed that although many people valued technology and felt it could support targeted activities, it was not seen as a substitute for traditional face-to-face therapy. Participants wanted technology to be more sophisticated and nuanced, supporting personalized and actionable recommendations. There was evidence that participants mistrusted technology, irrespective of the type, and had broader concerns regarding the impact of overuse of technology.

**Conclusions:**

People use different types of technology to support their mental health. Each can serve a specific purpose. Although apps are the most widely used technology, mixing and matching different types of technology is also common. Technology should not be seen as a replacement for traditional psychotherapy, rather it offers new opportunities to support mental health as part of an overall ecosystem. People want technology to be more nuanced and personalized to help them plan informed actions. Future interventions should explore the use of multiple technologies and their combined effects on mental health support.

## Introduction

### Background

Given the prevalence of mental health issues, such as depression, anxiety, and stress, there is an urgent need to help people manage their mental health. Stress, for example, accounts for 37% of all work-related ill health cases and 45% of all working days lost because of ill health in the United Kingdom [1], whereas depression alone affects 98.7 million people worldwide [2]. With increasing health care costs and limited access to treatment, technology-enabled solutions—ranging from systems designed to support therapy to apps and websites focused on mental health and well-being self-management—offer an affordable and accessible alternative. A wide range of studies have explored the impact, benefits, and limitations of individual technology types. With 87% of adults in the United Kingdom [3] and 89% of adults in the United States [4] having access to the internet, the potential for Web-based mental health systems has been recognized for several decades.

In the context of treatment, structured Web-based programs, particularly computerized Cognitive Behavioral Therapy (CBT) targeting depression and anxiety, have been widely researched. Examples include Beating the Blues [5], MoodGYM [6], Big White Wall [7], and SilverCloud [8]. Web-based interventions address the need to widen the access to treatment and research suggests that they can be effective [9-12], but challenges remain. For example, they tend to have short-term effects [13] and only when supported by a therapist, rather than as stand-alone interventions [14,15]. Moreover, low uptake rates indicate lower acceptability than noncomputerized approaches [16]. Poor adherence has been reported for Beating the Blues and MoodGYM in real-world settings [14]. The use of generic content rather than targeted content and absence of human support can make Web-based interventions feel impersonal [9,17].

Complementing treatment-focused programs, people can also access many websites and Web-based materials that support self-management of common mental health issues. For example, SuperBetter [18] encourages people to complete tasks that help them take care of their mental well-being and provides interactive content to keep them motivated; an evaluation study conducted with 283 participants suggests that it could be effective at helping people manage their depression [19]. Headspace [20] is another example, focused specifically on mindfulness. Although caution is required and further research is needed to more rigorously validate its efficacy in supporting different mental health difficulties, initial research suggests that people have positive attitudes toward it and that it might be effective [21]; however, many find it difficult to fit the use into their routines [22].

Building on evidence that peer support can have a positive impact on people’s mental health and well-being [23-25], Web-based peer support has become more common. Evidence suggests that the incorporation of moderated peer support can enhance Web-based treatment for early stage psychosis, with benefits including empowerment and increased social connectedness [26,27]. A study of 7Cups, a Web-based community focused specifically on mental health, provides preliminary evidence that people in emotional distress find support from trained nonprofessionals helpful [28]. More broadly, people can connect with others through general purpose social media sites, including Twitter and Facebook, and via discussion forums, such as Reddit, which has subreddits specifically focused on mental health issues. Evidence suggests people use these platforms to talk about mental health, as it gives them a sense of community, helps to raise awareness and fight stigma, provides a safe space for expression, and helps with coping and empowerment [29-31]. Although there is some research on the negative impact of social media on mental health, such as potential exacerbation of experiences associated with psychosis, mood disorders, and other mental illnesses [29], other studies suggest that social media is not a cause of mental health difficulty in and of itself [32], and that it can have positive impact in specific groups. For example, a case study by Veretilo and Billick [33] indicates that social networks may act as a springboard for greater societal integration, whereas Simoncic et al [34] suggest that Facebook use may be protective against depressive symptoms for female users with high levels of neuroticism.

Smartphone apps are the final category of technologies we address in this paper. Although social media and Web-based communities, such as Twitter or Facebook groups, are commonly accessed using smartphone apps, recent years have also witnessed a rapid increase in apps that specifically target mental health and well-being [35]. A number of recent systematic reviews and studies have analyzed the functionality offered by publicly available mental health apps [36-46], the context in which they are used and users’ experience [44], how people discover and choose apps [47], and apps’ grounding in theory [41-43,46]. Others have addressed the use of apps by specific population groups [48,49]. Although evidence suggests that a majority of publicly available apps are not grounded in theory, and this is a significant cause of concern, studies also suggest that appropriately designed apps can be effective in helping people address issues, including depression, stress, and anxiety [38,50,51].

### Objectives

Taken together, the literature outlined above has allowed the research community to build an understanding of how people use different mental health technologies, identifying strengths, limitations, areas of concern, and recommendations for improvement. However, studies have tended to focus on a single type of technology, for example, apps or Web-based systems. In reality, individual technologies may not be used in isolation.

To build a more complete picture of the real-world technology use, this paper investigates which technologies (smartphone apps, discussion forums or social media, and websites or Web-based programs) people use to support their mental health and why, whether they combine multiple technologies and, if so, what purpose each serves, and which features they find the most valuable.

## Methods

### Study Design and Recruitment

We conducted an anonymous online survey to explore the use of different types of technology to support mental health and well-being. People were asked to participate if they currently used or had previously used apps, social media, or websites for this purpose. The survey was open from February 2017 to May 2017. We neither actively sought nor excluded people who were experiencing mental health problems at the time of the survey. To reach a wide range of participants likely to use technology, the survey was advertised on social networks (Twitter and Facebook, where we used snowball sampling [52] by requesting retweets and shares from both participants and nonparticipants) and via posters distributed at university. As an incentive for completing the study, participants were entered into a raffle with a chance to win one of several shopping vouchers: a £30 voucher, 1 of 3 £20 vouchers, or 1 of 5 £10 vouchers. The study was approved by the Faculty of Engineering Research Ethics Committee at the University of Bristol, project ID: 48021.

### Survey Items

The survey comprised 59 questions, divided into 4 main sections: (1) the use of smartphone apps to support mental health and well-being, (2) the use of discussion forums or Web-based social networks to support mental well-being, (3) the use of websites or Web-based programs that offer support for mental well-being, and (4) background information about participants. In designing the survey, we recognized that technology categories used were not strictly exclusive, as, for example, it is not unusual for services like Reddit, Facebook, or Headspace to be accessed in multiple ways, including through a website or app. Our aim was to capture the use of broad categories of technology rather than specific products, while avoiding the creation of overly specific categories that would have resulted in an excessively long survey. For the purposes of this survey, we defined websites and Web-based programs that support mental health as sites that teach users skills and help them manage their well-being, for example, requiring more than a single visit and some input from the user. This includes websites that give users specific tasks to complete (eg, SuperBetter), Web-based programs that help to deal with stress or anxiety, support reflection on one’s behavior, and help create action plans (eg, MoodGYM), or websites that help users do specific things (eg, meditation websites, such as Headspace). Questions from sections 1 to 3 covered usage patterns, frequently used features, engagement with technology, and suggestions for improvements. At the end of the survey, participants were also able to opt in for the voucher raffle and indicate their interest in receiving the summary of findings. We collected email addresses of those who opted in; they were stored separately from the survey data. Survey questions and full results are available in Multimedia Appendices 1-3.

### Data Analysis

We used descriptive statistics to summarize general trends. In sections 1 to 3, survey respondents were able to provide suggestions for improvements and additional comments about each type of technology. We thematically coded these free-text comments and suggestions, and analyzed them together to identify wider themes spanning across all types of technology. The first round of coding was done by KS. Next, CP and DC reviewed the coding and added their own codes. Then, all authors discussed all codes and conducted affinity mapping [53], using Boardthing’s collaborative Web-based board [54] to identify key themes. For open-ended questions asking about frequency of use, KS initially coded the responses, and other coauthors reviewed them and provided their own suggestions; we then counted the prevalence of each code and reported percentages.

## Results

### Demographics and Technology Use

In total, 102 people completed the survey. Although the survey was targeted at people who used technology to support mental health, 21 participants (20.5%) indicated that they did not use technology for this purpose, with the main reason being that it never occurred to them to do so, they did not need it, or because they would not feel comfortable using technology. These participants were excluded from further analyses, leaving 81 participants whose responses we analyzed (see Table 1 for their details).

A total of 52% (42/81) of the participants were women, and 66% (53/81) of the participants were aged under 35 years. A majority of eligible participants (64/81, 79%) reported receiving some sort of mental health or well-being support from a counselor, therapist, or health professional. Within this group, 67% (43/64) of the respondents provided optional details, which showed that they had received counseling (23 participants) and CBT (11 participants); 22 participants mentioned a mix of these and other approaches (eg, workshops or medications). Although 45% of the participants (36/81) reported combining multiple technologies, 56% (45/81) of the participants indicated using only 1 category. Smartphone apps were the most commonly used technology, with 40% of the participants (32/81) using only them. Furthermore, 38% (31/81) of the participants reported using apps in combination with other technologies. Only 1 participant reported using websites or Web-based programs in isolation, and 15% of the participants (12/81) reported combining all categories of technology surveyed. Key findings related to each technology type are described in the following sections. The first 3 subsections focus on closed survey questions related to smartphone apps, forums or social media, and websites or Web-based programs, respectively. Additional usage trends are available in Multimedia Appendices 1-3. Free-text responses are addressed separately, and the results of the thematic analysis are reported in the final Results section.

**Table 1:** Demographics of participants who reported using technology to support their mental health and well-being and the types of technology used (N=81).

Demographics and technology use		Participants, n (%)
**Age (years)**		
	18-24	28 (35)
	25-34	25 (31)
	35-44	19 (24)
	45-54	7 (9)
	55-64	2 (3)
	65-74	0 (0)
	75+	0 (0)
**Gender**		
	Female	42 (52)
	Male	33 (41)
	Nonbinary	3 (4)
	Prefer not to say	3 (4)
**Received professional mental health support**		
	Yes	64 (79)
	No	15 (19)
	Prefer not to say	2 (3)
**Used only 1 type of technology**		
	Only smartphone apps	32 (40)
	Only social media or forums	12 (15)
	Only websites or Web-based programs	1 (1)
**Used multiple types of technology**		
	Apps and social media or forums	9 (11)
	Apps and websites or Web-based programs	10 (12)
	Social media/forums and websites/Web-based programs	5 (6)
	Apps and social media/forums and websites/Web-based programs	12 (15)

### Use of Smartphone Apps to Support Mental Well-Being

A total of 78% of the participants (63/81) reported using smartphone apps to support their mental well-being, either at the time of the survey (41/81, 51%) or in the past (22/81, 27%). In this section, we describe key findings only; all survey responses describing usage trends of apps for mental health support are available in Multimedia Appendix 1. Participants most often mentioned Headspace, a mindfulness meditation app: its use was reported by 41% (26/63) of the participants. Calm, another app providing meditation and relaxation content, was mentioned by 8 participants. Moodscope, a mood tracker, was the third most popular, with mentions from 4 participants. Details of the remaining 8 apps (each mentioned by 2 or 3 participants) are available in Multimedia Appendix 4. The main reasons participants reported for starting to use the apps were personal recommendations by someone known to them (20/63, 32%) and finding the app in the app store (also 32%) or on the internet (17/63, 27%). Only 4 participants (6%) reported that a health professional recommended the app.

A majority of participants (36/63, 57%) reported that the apps they had used provided guided activities, such as meditation or breathing exercises, or helped with relaxation through imagery or calming music (21/63, 33% responses). Features that allow tracking various factors related to well-being, for example, mood, thoughts, and sleep patterns, were mentioned by a third of the participants (22/63, 35%). Unsurprisingly, the features that participants considered most important largely corresponded to the features they used most often. When asked to select up to 3 types of features most important to them, a majority of participants selected guided activities (37/63, 59%), recording and tracking information (25/63, 40%), and relaxation (20/63, 32%). Table 2 shows types of functionality available in the apps used by survey respondents (as reported by them) and the features they reported as being the most valuable.

**Table 2:** Types of functionality available in the apps described by survey respondents versus types of functionality they reported as the most important to them, sorted by importance. Respondents were able to select up to 3 most important features (N=63).

Functionality	Respondents reporting that these features were available in the app they used, n (%)	Respondents listing these features as most important, n (%)
Guided activities (eg, meditation, breathing exercises, and mindfulness)	36 (57)	37 (59)
Recording and tracking things (eg, mood, thoughts, sleep patterns, exercise, and activities)	22 (35)	25 (40)
Relaxation (eg, imagery, calming music)	21 (33)	20 (32)
Self-reflection and understanding (eg, through a journal or a behavior and thought analysis)	13 (21)	18 (29)
Advice or recommendations for activities	10 (16)	16 (25)
Setting goals and planning activities	11 (18)	13 (21)
Information about mental well-being and reading materials	12 (19)	10 (16)
Social features (eg, chat, forums, and discussion groups)	8 (13)	6 (10)
Other^a^	0 (0)	2 (3)
Games	0 (0)	1 (2)
Tests and quizzes	2 (3)	0 (0)

^a^Includes “not sure” and “ability to visualize issues.”

Participants had an option to explain why they found these specific features the most important, and 68% (43/63) of them did so. Those who selected guided activities, such as meditation or breathing exercises, explained that these features helped them focus and deal with panic attacks, and they provided support “in the moment.” They also allowed them to develop regular daily routines and practice new skills. Recording and tracking were valued by participants because they provided insights into patterns of behavior, helped to quantify moods, and generally helped to keep track of progress and assess whether their behavior was changing. Participants for whom relaxation features were important, valued their help in dealing with stress and calming down before sleep. Previous research has suggested that factors including customization, interactive features, peer support, and input from professionals may be important in supporting engagement with mental health technologies [55]. When asked about their attitudes to these types of features available in the apps, participants reported that all except peer support were important or very important to them (see Table 3).

A majority of participants reported using the apps at least once per week (43/63, 68%), with 32% (20/63) of the participants using them daily. Others reported using apps for a limited period (7/63, 11%), with frequency of use decreasing over time (4/63, 11%). A few participants (4/63, 11%) reported using the apps only when needed (eg, when stressed or unable to sleep). When asked how they remembered to use the app, 48% (30/63) of the participants said they did it in response to events or sensations (eg, to stop a panic attack or reduce anxiety). In addition, 29% (18/63) of the participants said it was a part of their daily routine, and 13% (8/63) of the participants reported using the app automatically; 32% (20/63) of the participants reported receiving reminders. Although a majority of the participants (41/63, 65%) reported using apps for more than 3 months at the time of survey, 35% (22/63) of the participants said that they had already stopped using them. The most common reason for abandoning the apps was getting bored (8/22 participants, 36%), finding a better way to support mental well-being (6/22, 27%), and not needing the app anymore (4/22, 18%).

**Table 3:** Respondents’ attitudes toward aspects of smartphone apps that can influence engagement with technology (N=63).

Feature	Importance, n (%)				
	Very important	Important	Neither important nor unimportant	Unimportant	Very unimportant
Customization	19 (30)	28 (44)	10 (16)	3 (5)	3 (5)
Input from professionals	20 (32)	24 (38)	14 (22)	3 (5)	2 (3)
Peer support	8 (13)	6 (10)	13 (21)	24 (38)	12 (19)
Practical exercises	23 (37)	29 (46)	5 (8)	5 (8)	1 (2)
Self-monitoring	20 (32)	22 (35)	15 (24)	3 (5)	3 (5)

### Use of Discussion Forums and Social Media to Support Mental Well-Being

A total of 47% of the participants (38/81) reported using discussion forums or social media. They most frequently mentioned Reddit (9/38, 24%) and Facebook (8/38, 21%). The full list of forums and social media sites mentioned by participants is available in Multimedia Appendix 4. Below, we summarize key findings; full findings are available in Multimedia Appendix 2.

Overall, when asked about the importance of being able to connect to others on the Web about factors that influence mental well-being, 61% (23/38) of the participants said it was important or very important. Participants reported using social media and discussion forums primarily to read other people’s posts to learn more (32/38, 84%), help them understand their own situation (27/38, 71%), ask for advice (21/38, 55%), discuss problems and stressful situations (18/38, 47%), and give advice to others (18/38, 47%).

Among the survey participants who reported visiting discussion forums and social media to support their mental well-being, there were many who did so on a regular basis. A total of 29% (11/38) of the participants reported visiting them at least once a day, and 47% (18/38) of the participants reported visiting them at least once a week. A total of 24% (9/38) of the participants said they did it automatically, and 21% (8/38) of the participants said it was a part of their daily routine. Others reported doing so infrequently (4/38, 11%) or only when needed (6/38, 16%). Nearly half of the participants (17/38, 45%) reported that they logged in when they had a question or wanted to discuss something, and 42% (16/38) of the participants said they did that in response to events or sensations (eg, to stop a panic attack or reduce anxiety). A total of 66% of the participants (25/38) reported still using the discussion forums and social media at the time of the survey. Many participants also reported using discussion forums and social media over extended periods, with 58% (22/38) of the participants using them for over 6 months and 40% (15/38) of the participants using them for over 1 year. In contrast, 13 participants reported stopping using the site before the study, most commonly as they did not think they needed it anymore (6/13).

### Use of Websites and Web-Based Programs to Support Mental Well-Being

A total of 35% (28/81) of the participants reported using websites and Web-based programs to support their mental well-being. However, it is important to note that only 1 participant used websites and Web-based programs in isolation, with all others using them in combination with apps or social media. A full list of websites and Web-based programs reported by the participants is available in Multimedia Appendix 4.

The sites mentioned most often were MoodGYM (5/28, 18%), Headspace (3/28, 11%), NHS Direct (2/28, 7%), and Big White Wall (2/28, 7%). Below, we describe the key findings; full usage trends are available in Multimedia Appendix 3. In total, 46% (13/28) of the participants who reported using a website or a Web-based program were still using the site at the time of the survey, whereas 54% (15/28) of the participants had stopped doing so. The most common reason for stopping was finding a better way to support their mental health (7/15, 47%) or not needing the site’s support anymore (4/15, 27%). Regardless of whether they still used the sites, a majority of participants (20/28, 71%) reported using them primarily to find out how to deal with stress, anxiety, etc on a daily basis. Other reasons included wanting to develop specific skills (13/28, 46%), curiosity (9/28, 32%), recommendations from friends and family (7/28, 25%), and recommendations from health professionals (6/28, 21%). Participants reported using the sites either for a very short time, less than 4 weeks (12/28 respondents, 43%) or a prolonged period, over 12 months (9/28, 32%). A similar contrast was found in the follow-up question regarding frequency of use, with nearly a third of the participants (8/28, 29%) reporting using the sites once a month or less often, whereas 25% (7/28) of the participants reported doing it at least once per week. Where sites were used, it was often in response to events or sensations (14/28, 50%), although 18% (5/28) of the participants reported it was a part of their routine. When asked about engagement features available on the websites or Web-based programs they used, a majority of participants reported that practical exercises (24/28, 86%) and content created or endorsed by health professionals (23/28, 82%) were important to them (see Table 4).

**Table 4:** Respondents’ attitudes toward features that can support engagement available on websites or Web-based programs (N=28).

Feature	Importance, n (%)				
	Very important	Important	Neither important nor unimportant	Unimportant	Very unimportant
Customization	6 (21)	13 (46)	5 (18)	4 (14)	0 (0)
Interactive features	6 (21)	9 (32)	8 (29)	5 (18)	0 (0)
Input from professionals	13 (46)	10 (36)	1 (4)	3 (11)	1 (4)
Practical exercises	14 (50)	10 (36)	3 (11)	0 (0)	1 (4)
Self-monitoring	6 (21)	14 (50)	3 (11)	3 (11)	2 (7)

### Analysis of Open-Ended Comments and Suggestions for Improvements

Participants had the option to submit additional open comments about using each type of technology and suggest improvements. In total, 70% (57/81) of the participants provided such comments, which resulted in 119 open-ended responses that were coded and analyzed thematically. Through the analysis, we identified 4 themes reported below and discussed in more detail in the Discussion section. As one of the questions asked for suggestions for improvements, it is likely that participants took a critical perspective toward technology, with the emphasis on identifying limitations. This is reflected in the results.

#### Theme 1: Technology Can Support Targeted Activities, but It Is Not a Substitute for Face-to-Face Therapy

For some participants, technology, regardless of its type, wholly satisfied targeted aspects of their mental health needs. For example, a participant stated the following:

All I need is a mobile journaling tool with a search function that allows me to tag and store and later look up topics - so it's perfect really!

P12

The same person also appreciated the social network she frequently read:

The wonderful thing about the internet is that it's full of supportive communities of people with problems that are similar to yours. I believe that no problem is completely unique (there are always people in the same boat) and searching online helps me to find others who have the same problem, have figured it out and have shared it. I am more interested in hearing other people's solutions, perspectives and strategies for problems rather than ranting.

P12

In contrast, many participants had had face-to-face mental health treatments in the past and compared technology with this experience. The comparison was often negative, suggesting technology was a second-best option:

I ultimately found myself being more engaged with a therapist face to face - I felt like I was able to challenge and interrogate the actual therapy more.

P97

Some participants reported using technology as an alternative when traditional services were not available because of lack of time, access, or financial constraints:

I started using online services due to a recent lack of face-to-face support recently. I would prefer face-to-face in general, as I find it more stimulating and helpful.

P22

Frustration was noted when participants perceived that technology was being “pushed” inappropriately as a substitute for professional help, as a means to reduce costs. This highlights the fact that many participants did not see technology as a substitute for face-to-face treatment:

[Big White Wall] is really not a substitute for a good counsellor or support group (although it is being pushed as such by the university and by the NHS).

P12

#### Theme 2: People Want Personalized and Actionable Data

Participants highlighted the effectiveness of technology at supporting the acquisition of new habits, as well as their desire to be able to track various aspects of their behavior:

This to me was a great way to help me do some more meditation that I was trying to incorporate into my daily routine.

P67

Reminders are one of the features that could support this, but participants’ reports highlighted mixed attitudes toward them and the need for more sophisticated support that leads to action:

Less reminders but more suggestions how to put it into a routine / when to use it.

P10

Customization was seen as valuable, but participants regularly wished for greater flexibility and targeted customization to ensure the information they gathered was more meaningful and personalized:

Change the mood tracking questions so that I can spot some variation in them. As it stood, I used the mood tracker for a while, but I always gave the same answers, so it wasn't worth the bother.

P51

There was also a strong desire that technology supports a more nuanced view of mental health:

Could take more account of grey areas when it comes to mental health. [...] Just cause [sic] I can do daily activities to some extent doesn't mean that I am having a good mental health day.

P52

As noted above, some participants had a tendency to contrast technology with face-to-face support, noting that face-to-face approaches provided greater flexibility:

Although there were personalised [sic] elements (eg setting your own goals), it didn't always cover what I needed to cover (things that were later teased out from face to face CBT).

P97

Finally, although many participants recognized the benefits of activities, such as tracking, there was a clear desire for technologies that could translate this into recommendations for actions that are concrete, targeted, and personalized:

I haven't come across any that seem to be effective, at least for me. Something combining tracking and action based specifically on the results of the tracking might be best.

P61

#### Theme 3: Trust is a Critical Factor Across Mental Health Technologies

The issue of trust came up on 3 different levels: trust in the system, trust in the content, and trust in the community. The lack of trust in the system (app, website, and forum or social network) was represented by users’ concerns about privacy and security:

My primary concern would be around the use of the data I disclosed, not just by the company but potentially by other users in the future.

P6

Issues with trust in the content were represented by comments highlighting the need for more evidence-based materials or more input from professionals, which was related to reliability of information that was available or could be collected through technology:

There needs to be evidence! So many apps out there; don't want to waste my time on stuff that's rubbish or just out to make companies money - especially not when I'm feeling low.

P81

Another participant reported the following:

[I would like] more direct influence and response from experts, and having all the posts responded to by experts.

P20

Participants also raised issues with reliability of advice, the need for moderation, and concerns that unmoderated content may make things worse or be dangerous:

Useful when moderated. Access to mental health info/experiences of others on Tumblr/Twitter as a teenager served to make my health worse.

P52

Another participant said the following:

Often the people who are most ready to provide advice are not the best people to be giving that advice.

P79

#### Theme 4: Concerns About Overuse of Technology

Some survey respondents highlighted concerns toward technology in a general sense. For some, overuse of technology was seen as an issue in itself, and some people remarked that they simply did not want to use computers or apps more:

I feel somewhat uncomfortable with needing technology to look after my mental health. I probably spend too much time on my computer already.

P22

Other participants expressed feeling uncomfortable using mental health apps and reported that mental health technologies had the potential to exacerbate their condition:

The app itself did what it was supposed to - it just wasn't enough for me. I think on some level I hoped it would 'fix' my anxiety (ridiculous, I know) and when it (obviously) didn't, I began to associate the app with anxiety and seeing the icon on my phone made me worry about needing to use it.

P4

In some cases, these concerns were the reason for nonuse or discontinued use:

I am concerned about the addiction potential of smartphones and doubt that apps can support mental wellbeing. In my humble opinion mental wellbeing can even be reduced by using smartphones.

P66

## Discussion

### Principal Findings

Our findings show that people use different types of technology to support their mental health, and each can serve a distinct purpose: apps allow people to follow guided activities and monitor their health, social media and forums enable the discussion of issues with others and getting advice, whereas websites are a source of information about how to deal with issues on a day-to-day basis and help people develop specific skills. Smartphone apps were the most commonly used technology, but many people also mix and match different technologies. Irrespective of the type of technology, we found evidence that expectations can play a key role in people’s experience of technology. Particularly, the attitudes toward technology can be negative if it is presented (or “pushed”) as a replacement for face-to-face support. Technology can play an important role in supporting targeted activities, but to maximize this potential, participants indicated a desire for more nuanced and personalized technologies, which recognize the gray areas of mental health and help them plan informed actions. Trust is a key factor in people’s attitudes toward technology. These issues are discussed in greater detail below and considered in relation to previous literature.

#### Expectations and the Role of Technology

In discussing key problems with current digital mental health research, Mohr et al [56] argue that it is a misconception to view mental health technologies as simply a new way of delivering traditional, evidence-based psychotherapy. They argue instead that technology has the potential to revolutionize mental health and open fundamentally new intervention models. Their argument is focused on the research community, but our data suggest that it can be extended to include people who use technologies to support their mental health. Many of our participants had past or current experience of face-to-face care. Our data suggest that those who viewed technology negatively often did so on the basis of comparisons with face-to-face psychotherapy. In some cases, people felt technology was being incorrectly “pushed” as a replacement for face-to-face treatment because of resource constraints in health care services. Frustration in this case is understandable. However, it might be addressed as part of a broader reframing of technology, where it is viewed not as a replacement for traditional services but as part of a new, broader ecosystem in which technology both complements and extends traditional approaches. In this framing, the expectations of technology are subtly different: it is no longer a second-best option; rather, it is a different option that offers people new opportunities to engage with their mental health. The onus for researchers then also shifts to understanding how technology can extend the overall mental health ecosystem, identifying the contexts and groups with whom different technologies are most effective, setting realistic expectations of technology, and communicating these new choices to end users. In this study, those who used technologies as an active choice—or used it as an adjunct to a traditional therapeutic relationship—had different expectations and a more positive experience of technology. Previous studies [9,57] also show that technology is viewed more positively when it matches the expectations of users and is an active choice. Viewed as a single option in an overall ecosystem, the choice to not use technology or limit its use will be a correct decision for some people. The challenge is to identify approaches that are effective and fit individual needs, irrespective of the medium.

#### Apps and Smartphones

Many participants in this study combined different technologies, but smartphone apps were clearly the most widely used technology, either in isolation or in combination with others. Given that smartphones are also a common access point for social media and Web-based services, they clearly represent a vital part of the ecosystem of mental health technologies. Our findings corroborate the results of recent work by Schueller et al [47], which focused specifically on app use. Features such as tracking, notifications, interactivity, and customization are identified as important in both studies. Privacy, again identified as important in both studies, is discussed in greater detail below. Personal recommendations were the leading factor in people’s decision to use an app in both studies. Among our participants, only 6% of them were recommended an app by a health professional. This is lower than that reported by Schuller et al, but it is consistent with their finding that informal sources are currently more important than formal sources in identifying mental health apps. Interestingly, although the numbers in this study are too low to support strong conclusions, our findings indicate that health professionals in the United Kingdom are currently more likely to recommend Web-based resources than apps. It is possible that health professionals are less confident in the efficacy of apps, but it may also be because of institutional support from Web-based systems or a lack of awareness of available apps. Our evidence supports Schueller et al’s recommendation for further engagement with health care providers to understand their needs and raise awareness of apps that are safe and efficacious.

#### Moving Toward Personalized and Actionable Recommendations

It is important to recognize that dropout is not unique to technology-based mental health. It is also high in traditional face-to-face approaches. To maximize engagement with mental health services, the challenge is to identify approaches that are effective and fit individual needs and preferences, irrespective of the medium. Our results suggest that the combined use of different technologies can serve many different needs, ranging from information provision and professional and peer support to tracking and targeted activities. A strength of digital technology, smartphones in particular, is its ubiquity and personal nature [58]. It means that users can access support in the moment, in response to feelings or circumstances, and engage with a therapeutic practice or material when needed. This availability also means that it has potential to support the establishment of habitual practices to benefit mental well-being [22,44], perhaps more so than traditional psychotherapy. However, our results point toward a key direction through which future technologies can enhance peoples’ experience.

Many current technologies allow people to collect data or provide guided activities—features people appreciate—but most fall short in providing actionable data that lead to a positive change. This limitation has also been highlighted by other recent research [59-61]. Hollis et al [61] particularly emphasize the benefit of actionable analytics, where people are supported to reflect on both past and potential future selves and more constructively analyze their data to inform and plan actions that can support emotional well-being. In a similar vein, Rooksby et al [62] note that existing self-tracking features do not reflect the complex way in which people tend to monitor their health: they often interweave multiple data sources, share data with others, and have different needs depending on their short- or long-term goals. This is reflected in our participants’ desire for technologies to provide a more nuanced view of mental health. Greater personalization and support for this more nuanced approach, linked to targeted, action-oriented recommendations, is a key challenge for future research. Promising initial work in this area has been carried out by Mohr et al [51,63], who developed IntelliCare: an eclectic suite of highly focused elemental apps that together provide a wide range of features, which can be combined and used as needed.

#### Trust and Validity

Trust is a founding principle of effective psychotherapy relationships. If future systems are to make more nuanced use of personal data and social features, trust will be a critical issue. The ability to access information and connect with others is a key strength of technology. It allows Web-based communities to form, where people can access and share personal stories and mutual support, when forming face-to-face versions would be difficult or impossible. Although participants recognized the value in this, they also shared concerns, including how and with whom data are shared, the need for evidence-based systems, and appropriate moderation. More broadly, this links to previously identified concerns about privacy [48], the unregulated nature of digital health technologies [64-66], and limited evidence-base of mental health apps [41-44,46], which can reduce trust in technology and lower user engagement [67]. Web-based information and advice—be it from peer support groups, websites, or services provided in apps—effectively come with an implicit warning, reduced trust. This in turn, even when the information or service is completely authentic, may erode people’s confidence in the effectiveness of technology. Conversely, there is a danger that people may place unwarranted trust in technology that is labeled as being offered by a “professional.” This is worrying, as systematic reviews of publicly available apps show that not all apps that mention the involvement of health professionals in their descriptions provide evidence-based features [44]. Schuller et al [47] argue that research is needed to help people identify valid, evidence-based, and trusted sources. Stawarz et al also argue that the responsibility in this area must extend beyond the research and regulatory bodies, with responsibility also resting with those who distribute technology, for example, app store owners [44].

### Limitations and Future Work

The survey was advertised on social media and via posters on a university campus in the United Kingdom, which is likely to have attracted younger, more tech-savvy respondents. Although our survey software used cookies to reduce the likelihood of multiple responses by the same participant, we cannot guarantee this. We also specifically recruited people with experience of using technology to support their mental health. As such, our data cannot be taken to reflect the attitudes of the general public in regard to such technology. However, although this may limit the generalizability of our sample, it helped us attract participants who used multiple technologies, which offers insights into how they complement each other. Generalizability is also limited because of the size of the sample, but the fact that many of our results corroborate those of other recent research increases our confidence in our findings. Future work should explore this mixing and matching of technology in more detail, especially given that Millennials and future generations are growing up with technology, which will have an impact on the types of mental health support they may seek.

### Conclusions

Our research suggests that although apps are the most widely used mental health technology, people also tend to combine different technologies to support their mental health. Each technology can serve a specific purpose. Participants valued social features and felt technology can support targeted activities, but they were also aware of the limitations of technology. Many compared it unfavorably with face-to-face therapy. Users want apps, social media, and websites providing mental health support to be more trustworthy, nuanced, and personalized; they want actionable data about their health and clear guidance. This is both a challenge and an opportunity, as the mix of technologies suggests people may not necessarily need an all-in-one solution. Therefore, future interventions should explore the use of multiple technologies and their combined effects on mental health support.

## Appendix

Multimedia Appendix 1 Supporting mental health with apps: usage trends.

Multimedia Appendix 2 Supporting mental health with social media and discussion forums: usage trends.

Multimedia Appendix 3 Supporting mental health with websites and Web-based programs: usage trends.

Multimedia Appendix 4 Full list of apps, forums or social media, and websites or Web-based programs listed by survey respondents.

## Abbreviations

CBT: Cognitive Behavioral Therapy
NIHR: National Institute for Health Research
