# Supplementary material for: Use of Smartphone Apps, Social Media, and Web-Based Resources to Support Mental Health and Well-Being: Online Survey
Source: JMIR Ment Health. 2019 Jul 12;6(7):e12546. doi: 10.2196/12546 (PMC6659390; doi:10.2196/12546)
Supplement: Multimedia Appendix 1 [file mental_v6i7e12546_app1.pdf]

Multimedia Appendix 1

Supporting mental health with apps: usage trends

**Have you ever used apps to help you support your mental wellbeing, e.g. to reduce stress, help with your depression and/or anxiety, or track your mood?**	**N=102 n (%)**
Yes	63 (61.8)
No	39 (38.2)

**Details of use of apps**	**N=63 n (%)**
How did you find the app?	
Found it on the internet	17 (27)
Found it in the app store	20 (32)
It was recommended by an app store	1 (2)
It was recommended by someone I know	20 (32)
It was recommended by a therapist, counsellor or health professional	4 (6)
It was mentioned in an article I read	11 (18)
It was mentioned on social media	8 (13)
Other	2 (3)
How frequently do you / did you use the app?[a]	
Multiple times per day	3 (5)
Once a day	17 (27)
Multiple times per week	14 (22)
Once a week	9 (14)
Multiple times per month	1 (2)
Once a month	1 (2)
Less often than once a month	2 (3)
Irregularly	1 (2)
More often at first, less often now	4 (6)
Only when needed	4 (6)
For a limited time	7 (11)
How do you remember / what does make you use the app that often? (select all that apply)	
I receive reminders from the app	20 (32)
I have my own reminders (e.g. in a separate reminder app)	4 (6)
I receive notifications when I don't use the app for a while	4 (6)
It's a part of my daily routine	18 (29)
I do it automatically	8 (13)
I do it in response to events or sensations (e.g. to stop a panic attack or reduce anxiety)	30 (48)
Other	3 (5)
For how long have you been using / did you use the app?	
1 week or less	2 (3)
1-4 weeks	7 (11)

1-3 months	13 (21)
3-6 months	18 (29)
6-12 months	9 (14)
Over 12 months	14 (22)
Do you/did you share with anyone any information you recorded in the app?	
Yes	7 (11)
No	56 (89)

[a]It was an open-ended question. Answers were categories by the authors and grouped into the categories summarised in the table.

**Data sharing**	**N=7**<br>**n (%)**
With whom do you / did you share the information you recorded in the app? (select all that apply)	
With friends	5 (71)
With family	1 (14)
With partner	3 (43)
With therapist, counsellor or health professional	1 (14)
Other	1 (14)

**Continued use**	**N=63**<br>**n (%)**
Do you still use the app?	
Yes	41 (65)
No	22 (35)
Do you / did you use any other apps for supporting your mental wellbeing in the past couple of years?	
Yes	24 (38)
No	31 (49)
I don't remember	8 (13)
Do you still use these other apps?	
Yes	13 (21)
No	11 (18)

Why did you stop using the app? (select all that apply)	**N=22**<br>**n (%)**
I don't need it anymore	4 (18)
It didn't have features I needed	2 (9)
I found a better app	0 (0)
I found a better/different way of supporting my mental wellbeing	6 (27)
I got bored	8 (36)
Other	5 (23)

Why did you stop using the other apps? (select all that apply)	**N=11**<br>**n (%)**
I don’t need it anymore	3 (27)
It didn’t have features I needed	4 (36)
I found a better app	1 (9)
I found a better/different way of supporting my mental wellbeing	3 (27)
I got bored	4 (36)
Other	4 (36)
