# Supplementary material for: Use of Smartphone Apps, Social Media, and Web-Based Resources to Support Mental Health and Well-Being: Online Survey
Source: JMIR Ment Health. 2019 Jul 12;6(7):e12546. doi: 10.2196/12546 (PMC6659390; doi:10.2196/12546)
Supplement: Multimedia Appendix 2 [file mental_v6i7e12546_app2.pdf]

Multimedia Appendix 2

Supporting mental health with social media and discussion forums: usage trends

**Have you ever used any discussion forums or online social networks to support your mental wellbeing, e.g. to ask for advice or discuss any issues?**	**N=102 n (%)**
Yes	38 (37.3)
No	64 (62.7)

**Details of use of social media and discussion forums**	**N=38 n (%)**
On what device or devices do you access this site? (select all that apply)	
On my phone	24 (63)
On my tablet	8 (21)
On a shared tablet	0 (0)
On my PC / laptop	26 (68)
On a shared PC / laptop	0 (0)
On a work machine	6 (16)
On a public machine (e.g. in a library)	0 (0)
Other	0 (0)
How do you / did you access this site?	
Through a web browser	31 (82)
Through an app	12 (32)
Other	2 (5)
Who contributes to this discussion forum / social network? (select all that apply)	
People I know offline	6 (16)
Anonymous strangers	29 (76)
Strangers who use their real names	13 (34)
Experts (e.g. coaches, therapists, trainers)	4 (11)
Health professionals	6 (16)
I don't know if any experts of health professionals are involved	8 (21)
Other	1 (3)
How frequently do you / did you use this discussion forum / social network? [a]	
Multiple times per day	2 (5)
Once a day	9 (24)
Multiple times per week	4 (11)
Once a week	3 (8)
Multiple times per month	1 (3)
Once a month	3 (8)
Less often than once a month	4 (11)
More often at first, less often now	1 (3)
Only when needed	6 (16)
For a limited time	5 (13)

How do you remember / what does make you visit this site that often? (select all that apply)	
I visit it regularly anyway, for reasons not related to mental wellbeing support	11 (29)
I visit it when I have a question or want to discuss something	17 (45)
I receive reminders from the site	1 (3)
I have my own reminders (e.g. in a separate reminder app)	0 (0)
I receive notifications when I don't use the site for a while	0 (0)
It's a part of my daily routine	8 (21)
I do it automatically	9 (24)
I do it in response to events or sensations (e.g. to stop a panic attack or reduce anxiety)	16 (42)
Other	7 (18)
For how long have you been using / did you use the app?	
1 week or less	3 (8)
1-4 weeks	3 (8)
1-3 months	4 (11)
3-6 months	6 (16)
6-12 months	7 (18)
Over 12 months	15 (40)

[a]It was an open-ended question. Answers were categories by the authors and grouped into the categories summarised in the table.

**Continued use**	**N=38 n (%)**
Do you still visit this social media site / discussion forum?	
Yes	25 (66)
No	13 (34)
Do you / did you use other discussion forums or social networks to support your mental wellbeing?	
Yes	6 (16)
No	29 (76)
I don't remember	3 (8)

Why did you stop visiting this site? (select all that apply)	**N=13 n (%)**
I don't need it anymore	6 (46)
I found a better/different way of supporting my mental wellbeing	2 (15)
I got bored	1 (8)
Other	4 (31)

Do you still visit these other social media sites or discussion forums?	**N=6 n (%)**
Yes	4 (67)
No	2 (33)

Why did you stop using the other sites? (select all that apply)	**N=2**<br>**n (%)**
I don’t need it anymore	0 (0)
I found a better/different way of supporting my mental wellbeing	1 (50)
I got bored	0 (0)
Other	2 (100)
