# Supplementary material for: Use of Smartphone Apps, Social Media, and Web-Based Resources to Support Mental Health and Well-Being: Online Survey
Source: JMIR Ment Health. 2019 Jul 12;6(7):e12546. doi: 10.2196/12546 (PMC6659390; doi:10.2196/12546)
Supplement: Multimedia Appendix 3 [file mental_v6i7e12546_app3.pdf]

Multimedia Appendix 3

Supporting mental health with websites and Web-based programs: usage trends

**Have you ever used websites or Web-based programs to help you support your mental wellbeing, e.g. to help you deal with stress, anxiety or depression?**	**N=102 n (%)**
Yes	28 (27.5)
No	74 (72.5)

**Details of use of websites and Web-based programs**	**N=28 n (%)**
Why did you decide to use this website or Web-based program? (select all that apply)	
To find out how to deal with stress, anxiety, etc. on a day-to-day basis	20 (71)
To develop specific skills or strategies	13 (46)
Out of curiosity	9 (32)
Because a friend or a family member suggested I should try it	7 (25)
Because it was recommended to me by a GP or another healthcare professional	6 (21)
As part of professional development	4 (14)
Other	1 (4)
How frequently do you / did you visit this website or Web-based program?[a]	
Multiple times per day	0 (0)
Once a day	5 (18)
Multiple times per week	1 (4)
Once a week	1 (4)
Multiple times per month	0 (0)
Once a month	3 (11)
Less often than once a month	5 (18)
Irregularly	3 (11)
More often at first, less often now	0 (0)
Only when needed	2 (7)
For a limited time	8 (29)
How do you remember / what does make you visit this site that often? (select all that apply)	
I get notifications when I don't use the site for a while	3 (11)
It's a part of my daily routine	5 (18)
I do it automatically	2 (7)
I do it in response to events or sensations (e.g. to stop panic attack or reduce anxiety)	13 (46)
Other	9 (32)
For how long have you been using / did you use the site?	
1 week or less	7 (25)

1-4 weeks	5 (18)
1-3 months	1 (4)
3-6 months	2 (7)
6-12 months	4 (14)
Over 12 months	9 (32)
On what device or devices do you / did you access this site? (select all that apply)	
On my phone	16 (57)
On my tablet	5 (18)
On a shared tablet	0 (0)
On my PC / laptop	16 (57)
On a shared PC / laptop	0 (0)
On a work machine	2 (7)
On a public machine (e.g. in a library)	0 (0)
Other	0 (0)
Does this site have a companion app?	
Yes	6 (21)
No	4 (14)
I don't know	18 (64)

[a]It was an open-ended question. Answers were categories by the authors and grouped into the categories summarised in the table.

Do you use the companion app?	**N=6 n (%)**
Yes	4 (67)
No	2 (33)

**Continued use**	**N=28 n (%)**
Do you still visit this website / Web-based program?	
Yes	13 (46)
No	15 (54)

Why did you stop using this website / Web-based program? (select all that apply)	**N=15 n (%)**
I don't need it anymore	4 (27)
I completed the program	2 (13)
I found a better/different way of supporting my mental wellbeing	7 (47)
I got bored	3 (20)
Other	3 (20)

Do you / did you use any other websites or Web-based programs to support your mental wellbeing?	**N=28 n (%)**
Yes	5 (18)
No	16 (57)

I don't remember	7 (25)

Do you still visit these other websites or Web-based programs ?	**N=5<br>n (%)**
Yes	3 (60)
No	2 (40)

Why did you stop using those websites or Web-based programs ? (select all that apply)	**N=2<br>n (%)**
I don't need it anymore	1 (50)
I completed the program	1 (50)
I found a better/different way of supporting my mental wellbeing	0 (0)
I got bored	0 (0)
Other	0 (0)
